# In Silico and In Vitro Studies of Anti-Inflammatory, Anti-Oxidative Stress, and Anti-Apoptosis Effect of 7-Octenoic Acid Derived from *Moringa oleifera* Lam., on LPS-Induced Monocyte-Derived Macrophages (MDM)

**DOI:** 10.3390/ijms26188911

**Published:** 2025-09-12

**Authors:** Kittipong Srimuang, Watunyoo Buakaew, Yordhathai Thongsri, Krai Daowtak, Pachuen Potup, Antonio Ferrante, Kanchana Usuwanthim

**Affiliations:** 1 Cellular and Molecular Immunology Research Unit (CMIRU), Faculty of Allied Health Sciences, Naresuan University, Phitsanulok 65000, Thailand; kittipongs65@nu.ac.th (K.S.); watunyoo@g.swu.ac.th (W.B.); yordhathait@nu.ac.th (Y.T.); kraid@nu.ac.th (K.D.); pachuenp@nu.ac.th (P.P.); 2 Department of Microbiology, Faculty of Medicine, Srinakharinwirot University, Bangkok 10110, Thailand; 3 Department of Immunopathology, South Australia Pathology, Women’s and Children’s Hospital, Adelaide, SA 5000, Australia; antonio.ferrante@adelaide.edu.au; 4 Department of Paediatrics, Adelaide School of Medicine, University of Adelaide, Adelaide, SA 5000, Australia; 5 Department of Molecular and Biomedical Science, School of Biological Science, University of Adelaide, Adelaide, SA 5000, Australia; 6 Robinson Research Institute, University of Adelaide, University of Adelaide, Adelaide, SA 5000, Australia

**Keywords:** inflammation, oxidant, apoptosis, bioactive compound

## Abstract

While *Moringa oleifera* Lam. (MO) extracts are known to have various bioactive properties, including anti-inflammatory properties, the components responsible still remain to be identified. This study explores the protective effects of the MO component, 7-octenoic acid (7OCT) in LPS-stimulated THP-1 macrophage inflammatory responses. The compound significantly downregulated the production of the pro-inflammatory cytokines TNF-α, IL-1β, and IL-6, as well as the expression of inflammation-related genes *NFKB1*, *PTGS2*, and *NOS2*. Additionally, it inhibited the nuclear translocation of NF-κB p65, a key transcription factor of inflammatory signaling cascade. Effects on oxidative stress showed that 7OCT inhibited LPS-induced NADPH oxidase 2 (NOX2) component genes including *CYBB*, *CYBA*, *NCF1*, *NCF2*, and *NFE2L2*, along with phosphorylated NOX2 and p47^phox^ proteins. The compound reduced the expression of *TP53*, *BAX*, *CASP3*, and *CASP7*, while enhancing *BCL2* expression and Bcl-2 protein levels, suggesting an effect on apoptosis. Decreased levels of BAX, caspase-3, and cleaved caspase-3 proteins further confirmed its anti-apoptotic effect. Our findings suggest that 7OCT exhibits strong anti-inflammatory, antioxidant, and anti-apoptotic properties.

## 1. Introduction

Inflammation is a crucial physiological process that enables the host to respond to infections and tissue injuries. However, when dysregulated, it leads to chronic debilitating diseases, including cancer, neurodegeneration, and cardiovascular disorders. One of the best-characterized triggers of pathogenic inflammation is lipopolysaccharide (LPS), a structural component of the outer membrane of Gram-negative bacteria. LPS functions as a pathogen-associated molecular pattern (PAMP) and activates innate immune responses through Toll-like receptor 4 (TLR4) on the surface of immune cells such as macrophages and dendritic cells. Upon LPS binding, TLR4 undergoes dimerization and recruits the adaptor protein MyD88, which initiates downstream signaling involving interleukin-1 receptor-associated kinases (IRAKs) and TNF receptor-associated factor 6 (TRAF6). This cascade leads to activation of the IκB kinase (IKK) complex, which phosphorylates IκBα, predisposing it for ubiquitination and subsequent proteasomal degradation. Freed from inhibition, NF-κB (primarily p65/RelA and p50 subunits) translocated to the nucleus and initiates transcription of pro-inflammatory cytokines such as TNF-α, IL-1β, and IL-6, as well as enzymes like inducible nitric oxide synthase (iNOS) and cyclooxygenase-2 (COX-2) [1,2,3,4]. This inflammatory response is tightly associated with the generation of reactive oxygen species (ROS) [5], which act as secondary messengers in redox signaling but also contribute to cellular damage when overproduced [5,6]. In exacerbated inflammation, excessive ROS can induce oxidative stress, disrupt cellular macromolecules, and perpetuate the inflammatory cycle [7,8].

One of the principal enzymatic sources of ROS in immune cells is the NADPH oxidase 2 (NOX2) complex, particularly in phagocytes like neutrophils and macrophages. NOX2 is a multi-component enzyme system that catalyzes the reduction of molecular oxygen (O_2_) to form superoxide anion (O_2_•^−^) using electrons donated from NADPH. The NOX2 complex comprises both membrane-bound and cytosolic components. The membrane-associated flavocytochrome b558 is composed of gp91phox (CYBB) and p22phox (CYBA), which form the catalytic core. In resting cells, the cytosolic regulatory subunits—p47phox (NCF1), p67phox (NCF2), p40phox (NCF4), and the small GTPase Rac1/2—remain inactive in the cytoplasm. Upon activation by LPS or pro-inflammatory cytokines, p47phox undergoes serine phosphorylation, allowing it to translocate and bind to p22phox via its SH3 domain, initiating assembly of the active oxidase complex at the membrane [9,10,11,12]. p67phox functions as the activator of electron flow, interacting with gp91phox to initiate superoxide production. p40phox modulates this process and may stabilize p67phox binding under certain conditions [13,14]. Rac GTPase plays a critical role in structural activation and signal integration. The resulting ROS, including superoxide and hydrogen peroxide, which contribute not only to microbial killing but also to redox-sensitive signaling, which can influence cell survival, inflammation, and apoptosis [15,16]. Dysregulation of NOX2 has been implicated in a range of inflammatory and degenerative diseases due to excessive ROS generation and oxidative tissue damage [17].

Under persistent oxidative stress or unresolved inflammation, cells may initiate apoptosis, a programmed cell death mechanism that maintains tissue homeostasis. One of the central regulators of this process is the tumor suppressor protein p53, which becomes stabilized and activated in response to DNA damage, oxidative stress, and oncogenic signals. Activated p53 transcriptionally upregulates several pro-apoptotic genes, notably Bax, which belongs to the Bcl-2 family of proteins. Bax is typically cytosolic, but upon activation, it undergoes conformational change, translocated to the mitochondrial outer membrane, and oligomerizes to form pores that facilitate the release of cytochrome c into the cytoplasm [18,19,20]. Once in the cytosol, cytochrome c binds Apaf-1, forming the apoptosome that recruits and activates procaspase-9. Active caspase-9 then cleaves and activates executioner caspases such as caspase-3 and caspase-7, which degrade intracellular substrates, leading to DNA fragmentation, membrane blebbing, and cell dismantling [21,22]. The balance between pro-apoptotic (e.g., Bax, Bak) and anti-apoptotic proteins (e.g., Bcl-2, Bcl-xL) is critical in determining cell fate. Bcl-2 opposes apoptosis by sequestering Bax and preventing mitochondrial permeabilization. Notably, ROS can modulate this balance by inducing p53 and suppressing Bcl-2 expression, pushing the cell toward apoptosis [23,24,25]. Aberrations in this tightly controlled system are implicated in cancer, neurodegeneration, and autoimmune disorders, highlighting the importance of the p53-Bcl-2 axis in cell death regulation. Inflammation and oxidative stress, which ultimately lead to cell death, remain key therapeutic targets, highlighting the ongoing need for novel agents to guide the development of new anti-inflammatory drugs.

The phytochemicals’ therapeutic potential has garnered considerable scholarly interest within anti-inflammatory, antioxidant and anti-cancer research, owing to plant-derived compounds’ demonstrated bioactivity across both in vitro and in vivo models [26,27,28,29,30,31,32]. *Moringa oleifera* Lam. (MO) is a taxonomic affiliation with the Moringaceae family, and its widespread cultivation across numerous Southeast Asian regions, most notably Thailand [33]. The plant’s pharmacological profile has made it a focal point of research across multiple phytomedicinal domains, owing to its anti-inflammatory [34,35], anticancer [36,37], antioxidant [38], and antimicrobial [39] effects. The study from Wisitpongpun et al. demonstrated that 7-octenoic acid (7OCT) enhances anticancer activity against breast cancer cells by inducing cell cycle arrest and promoting apoptosis [40]. However, investigations into 7OCT’s anti-inflammatory, antioxidative, and anti-apoptotic activities remain largely unexplored.

In this study, we investigated the effects of 7OCT on macrophage inflammatory, oxidative stress, and apoptosis response. The gene and protein expression levels were measured for all the studied targets. Furthermore, molecular docking was performed to predict the interactions between 7OCT and proteins predicted to be involved in inflammation, oxidative stress, and apoptosis. Our results show that 7OCT significantly downregulated the expression of pro-inflammatory cytokines TNF-α, IL-1β, and IL-6 and inhibited LPS-induced NADPH oxidase 2 (NOX2) along with reduced the expression of *TP53, BAX, CASP3*, and *CASP7*. These provide valuable insights into the anti-inflammatory, antioxidant, and anti-apoptotic activities of 7OCT found in Moringa leaves, which helps to advance our understanding of the therapeutic and health-promoting potential of this compound.

## 2. Results

### 2.1. The Effect of 7-Octenoic Acid (7OCT) on Cell Viability

**Figure 1:**
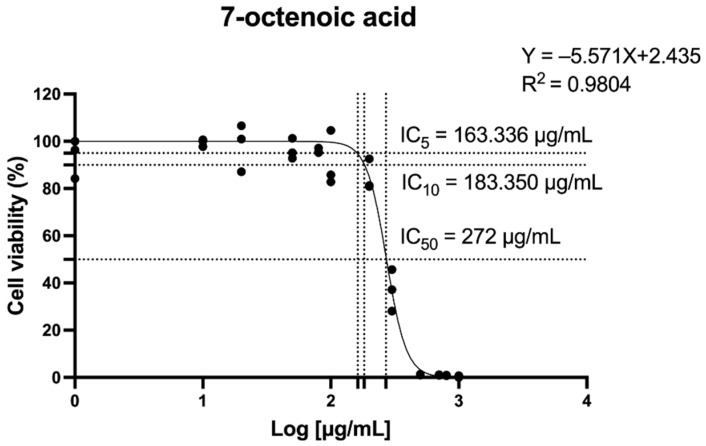
The 7-octenoic acid dose–response curves of monocytic THP-1 cell viability. Data represent the mean ± SEM of three independent experiments. IC50, the half-maximal inhibitory concentration.

The cytotoxic profile of 7OCT was assessed in THP-1-derived macrophages following a 24-h exposure to varying concentrations of the compound, with cell viability evaluated using the MTT assay. The 7OCT dose–response relationship was determined through nonlinear regression analysis using GraphPad Prism 10.0 (Figure 1). The IC_5_ and IC_10_ values were computed from standard sigmoidal dose–response equations, where F denotes the target inhibition percentage and H corresponds to the Hill slope. The experimentally derived IC_5_, IC_10_, and IC_50_ values for 7OCT were found to be 160.336 µg/mL, 183.350 µg/mL, and 272 µg/mL, respectively. For further study, 7OCT concentrations of 50, 80, and 100 μg/mL were selected.

$${IC}_{F}={\left(\frac{F}{100-F}\right)}^{\sqrt{H}}\cdot {\mathrm{IC}}_{50}$$

### 2.2. Prediction of Interactions Between 7OCT and Protein Target Using Molecular Docking Analysis

In this study, molecular docking was utilized to predict the binding potential of 7OCT with key protein targets associated with inflammation, oxidative stress, and apoptosis, using Auto Dock Vina version 1.2.3, with further 2D and 3D interaction visualization conducted via BIOVIA Discovery Studio Visualizer version 21.1.0.20298. The ligands against each receptor protein were shown in ligand-protein interactions through active amino acids of the catalytic pockets and free energy structures (Table 1). The results demonstrated that 7OCT exhibited favorable binding affinities with inflammation-related proteins, including TLR4, RelA, IκB-α, p50, and COX-2, with binding energies ranging approximately from –3.7 to –5.5 kcal/mol. For oxidative stress-related proteins such as NOX2 and its associated subunits (p22phox, p40phox, p47phox, and p67phox), 7OCT showed binding values between –5.9 and –4.0 kcal/mol, indicating the compound’s possible role in inhibiting NADPH oxidase activity, which could lead to reduced reactive oxygen species (ROS) production. Additionally, interactions with apoptotic regulators, including BAX, Caspase-3, Caspase-7, and Bcl-2, with binding affinities ranging from −5.0 to 4.2 kcal/mol.

**Figure 2:**
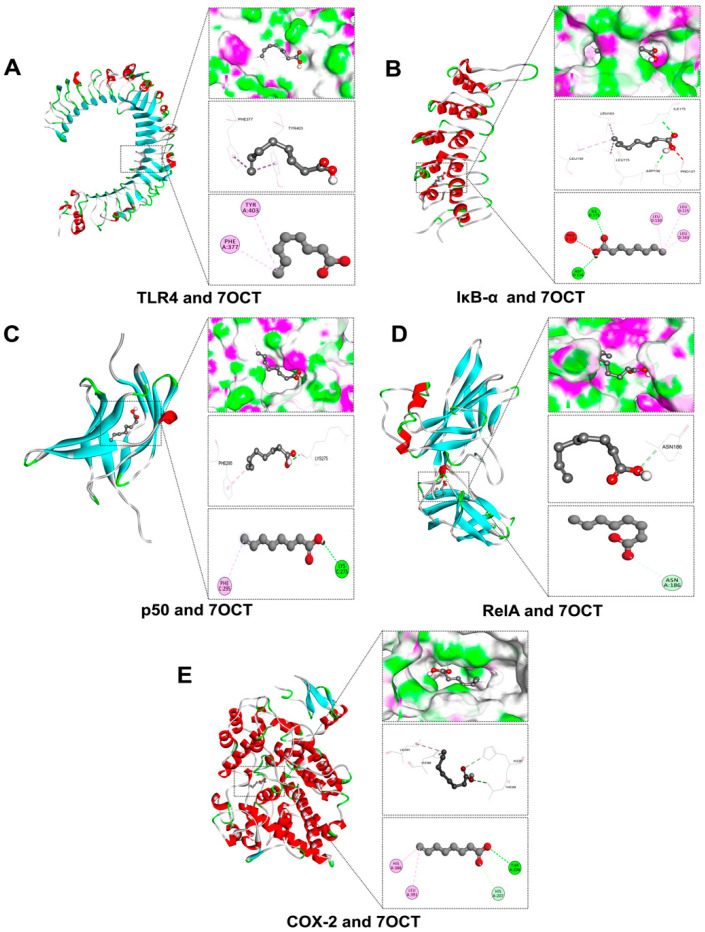
Molecular docking of 7OCT between inflammatory proteins: TLR4 (**A**), IκB-α (**B**), p50 (**C**), RelA (**D**), and COX-2 (**E**).

In this in silico study, the interaction of 7OCT with inflammation-associated proteins was analyzed using molecular docking and visualized through BIOVIA Discovery Studio Visualizer version 21.1.0.20298 to predict its anti-inflammatory potential at the molecular level (Figure 2). The docking simulations revealed that 7OCT exhibited a relatively weak binding affinity with TLR4 (–3.7 kcal/mol) (Figure 2A), interacting specifically with PHE377 and TYR403 via Pi-alkyl interactions, suggesting a modest engagement at the receptor level, potentially affecting early innate immune signaling. A stronger interaction was observed with RelA (–5.0 kcal/mol) (Figure 2D), a critical transcription factor in the NF-κB complex, where 7OCT formed a carbon–hydrogen bond with ASN186, indicating possible disruption of DNA-binding or nuclear translocation activity. For IκB-α (–4.6 kcal/mol) (Figure 2B), which inhibits NF-κB in the cytoplasm, 7OCT established conventional hydrogen bonds with ASP136 and ILE175, alkyl interactions with LEU115, LEU130, and LEU163, and an unfavorable acceptor–acceptor interaction with PRO137, reflecting a complex and potentially destabilizing influence on IκB-α’s structural integrity, which may modulate NF-κB activation. In the case of NF-κB p50 (–4.5 kcal/mol) (Figure 2C), interaction occurred via a conventional hydrogen bond with LYS275 and a Pi-alkyl bond with PHE295, implying a possible hindrance in p50 dimerization or DNA-binding functions. Notably, the most favorable binding among all inflammatory targets was observed with COX-2 (–5.5 kcal/mol) (Figure 2E), where 7OCT formed a network of interactions including a carbon–hydrogen bond with HIS207, Pi-alkyl and alkyl bonds with HIS388 and LEU391, and a conventional hydrogen bond with THR206. These findings collectively suggest that 7OCT possesses multi-site binding capability across several key components of the inflammatory pathway, with its strongest inhibitory potential likely at the level of COX-2 and NF-κB signaling modulators, which may translate into downstream suppression of pro-inflammatory gene expression and mediator production.

**Figure 3:**
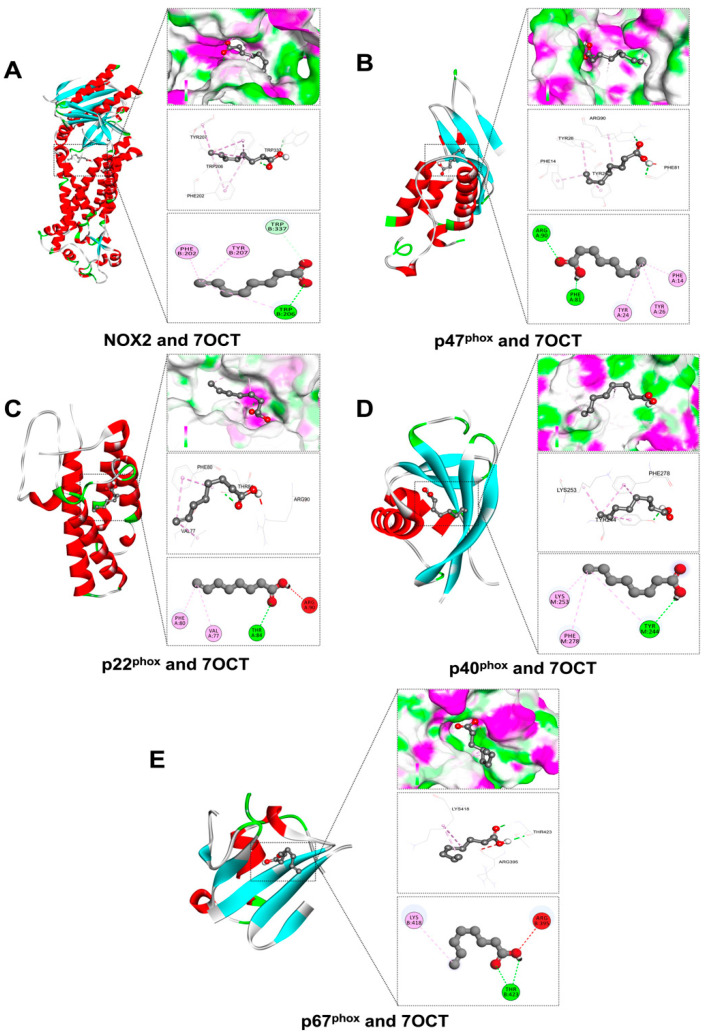
Molecular docking of 7OCT between oxidative stress-related proteins: NOX2 (**A**), p47^phox^ (**B**), p22^phox^ (**C**), p40^pho^x (**D**), and p67^phox^ (**E**).

The interaction of 7OCT with key subunits of the NADPH oxidase (NOX2) complex, a major enzymatic generator of reactive oxygen species (ROS), was examined to elucidate its potential antioxidant mechanism, using BIOVIA Discovery Studio Visualizer version 21.1.0.20298 (Figure 3). The docking results revealed that NOX2 exhibited the strongest binding affinity (–5.9 kcal/mol) with 7OCT (Figure 3A), where TRP207 formed a conventional hydrogen bond and TRP337 formed a carbon hydrogen bond, implying a potentially stable interaction at the catalytic core, which may interfere with electron transport during ROS generation. For p47^phox^, a critical cytosolic organizer protein involved in NOX2 activation, 7OCT displayed substantial affinity (–5.4 kcal/mol) (Figure 3B), forming carbon hydrogen bonds with ARG90 and PHE81, and Pi-alkyl interactions with PHE14, TYR24, and TYR26, suggesting its ability to disrupt p47phox conformational shifts necessary for membrane translocation and complex assembly. Interaction with p22^phox^ (–4.9 kcal/mol) (Figure 3C), a membrane-bound stabilizer of NOX2, revealed both unfavorable donor–donor bonding with ARG90 and stabilizing interactions through conventional hydrogen bonding with THR84 and Pi-alkyl/alkyl contacts with PHE80 and VAL77, indicating a mixed stabilizing–disruptive profile. Docking with p40^phox^ (–4.2 kcal/mol) (Figure 3D) showed interaction with TYR244 via a conventional hydrogen bond, and LYS253 and PHE27 via Pi-alkyl and alkyl bonds, highlighting a moderate affinity that may alter its regulatory function within the complex. Finally, 7OCT showed the weakest affinity for p67^phox^ (–4.0 kcal/mol) (Figure 3E), where ARG395 participated in an unfavorable acceptor–acceptor interaction, while THR423 and THR206 formed conventional hydrogen bonds, and LYS418 engaged through alkyl bonding, suggesting a limited but specific interaction at regions potentially involved in Rac binding or activation. Together, these findings illustrate 7OCT’s preferential binding to NOX2 and p47phox, with the potential to interfere with both enzymatic activity and cytosolic subunit recruitment, thereby disrupting ROS production at multiple levels.

**Figure 4:**
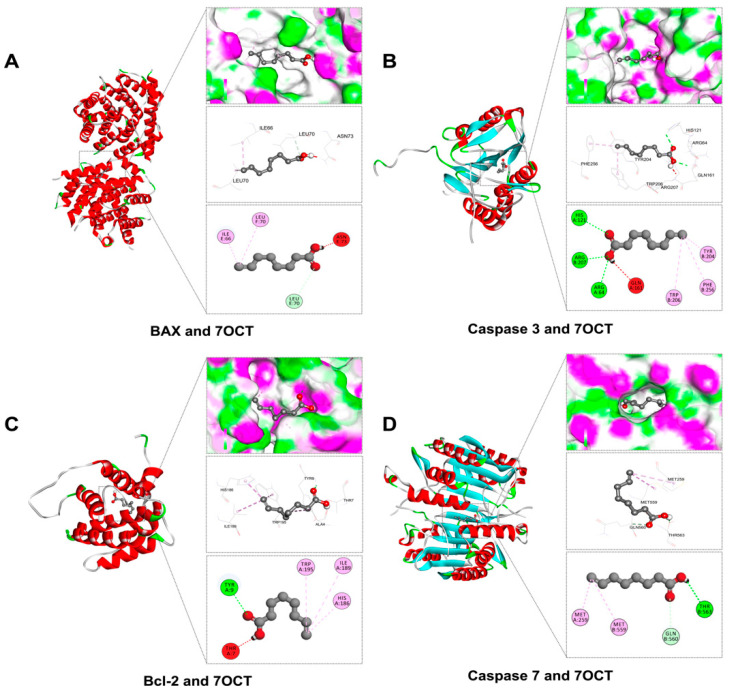
Molecular docking of 7OCT between apoptotic regulators: BAX (**A**), Caspase 3 (**B**), Bcl-2 (**C**), and Caspase 7 (**D**).

The molecular docking evaluation of 7OCT against apoptosis-regulating proteins provided mechanistic insights into its potential modulatory role in cell death pathways, analyzed using BIOVIA Discovery Studio Visualizer version 21.1.0.20298 (Figure 4). The compound demonstrated a binding affinity of –5.0 kcal/mol with BAX, a pro-apoptotic member of the Bcl-2 family, forming alkyl interactions with ILE66 and IEU70, while an unfavorable donor–donor interaction with ASN73 could potentially destabilize the BAX conformational structure. In the case of Caspase-3, an executioner protease central to apoptotic signaling, 7OCT exhibited a binding affinity of –4.7 kcal/mol through multiple conventional hydrogen bonds with ARG64, HIS121, and ARG207, accompanied by Pi-alkyl interactions with PHE256, TRP206, and TYR204, although the presence of an unfavorable donor–donor bond with GLN161 may interfere with optimal binding. For Caspase-7, which shares structural and functional homology with Caspase-3, 7OCT bound with lower affinity (–4.2 kcal/mol), forming carbon hydrogen and conventional hydrogen bonds with GLN560 and THR563, and engaging MET259 and ME559 through alkyl bonds, possibly affecting substrate recognition or cleavage efficiency. Lastly, 7OCT interacted with Bcl-2, an anti-apoptotic protein, at a binding energy of –4.5 kcal/mol, forming a conventional hydrogen bond with TYR9, an unfavorable donor–donor bond with THR7, and stabilizing Pi-alkyl and alkyl interactions with HIS186, ILE189, and TRP195, which may interfere with its anti-apoptotic activity by altering the hydrophobic groove critical for BAX/BAK binding. Collectively, these results suggest that 7OCT can bind both pro- and anti-apoptotic proteins with moderate affinity, potentially modulating the intrinsic apoptotic pathway and influencing cell survival dynamics.

### 2.3. The Effect of 7OCT on the Expression of Pro-Inflammatory Cytokine Production in LPS-Stimulated Macrophages

**Figure 5:**
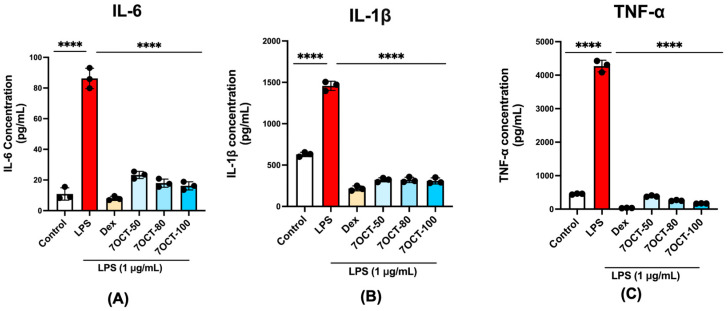
Effect of 7OCT on pro-inflammatory cytokine production in LPS-stimulated THP-1-macrophages. Panels (**A**–**C**) illustrate the levels of IL-1β, IL-6, and TNF-α, respectively, in macrophages stimulated with 1 μg/mL LPS following treatment with the drug (Dex) and 7OCT at concentrations of 50, 80, and 100 μg/mL. Results represent the mean ± SEM of three independent experiments (*n* = 3); **** *p* < 0.0001 compared to control, **** *p* < 0.0001 compared to LPS. Control: Untreated THP-1 macrophage; LPS: Lipo-polysaccharide-stimulated THP-1 macrophage; Dex: Dexamethasone; 7OCT: 7-Octenoic acid.

To assess the anti-inflammatory efficacy of 7OCT, we quantified the concentrations of key pro-inflammatory cytokines IL-6, IL-1β, and TNF-α in the culture supernatants of differentiated macrophages subjected to treatment with the compound. Following the development of macrophages, cells were treated with lipopolysaccharide (LPS) after being pre-incubated with 7OCT for an hour. Twenty-four hours after incubation, the amounts of secreted cytokines were assessed. LPS caused a significant increase in the levels of IL-6, IL-1β, and TNF-α production by the macrophages (Figure 5). Pretreatment of cells with 7OCT or the standard anti-inflammatory agent resulted in a significant decrease in production of these cytokines, indicating that the compound has effective anti-inflammatory activity judged by this criterion.

### 2.4. The Effect of 7OCT on the NF-κB Pathway in LPS-Stimulated Macrophages

**Figure 6:**
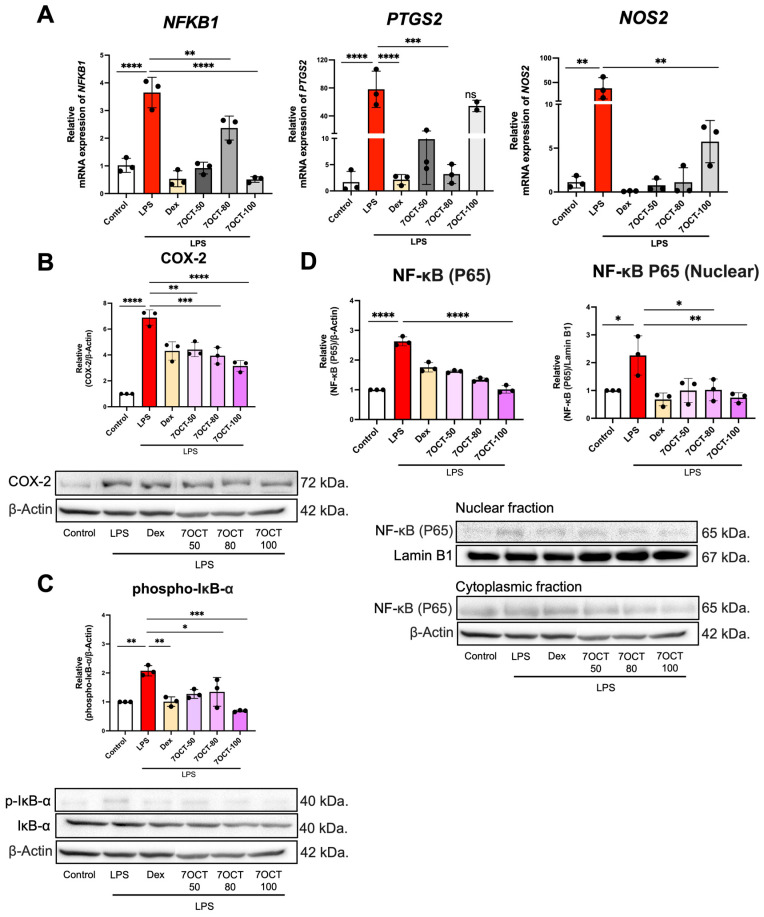
The effect of 7OCT on the mRNA expression of pro-inflammatory markers (**A**) and the protein expression levels of NF-κB (p65), phosphorylated IκB-α, and COX-2 in LPS-stimulated macrophages. Western blot analysis was performed to assess the expression of these proteins (**B**,**C**), and the relative nuclear p65 protein levels (**D**) were quantified by densitometric scanning and normalized against the control group. Lamin B1 were employed as internal controls for nuclear and β-actin were employed as internal controls for cytoplasmic protein normalization. Results represent the mean ± SEM of three independent experiments (*n* = 3). A value of *p*  <  0.05 was considered to be significant. Significant differences between groups are marked with: * *p*  <  0.05, ** *p*  <  0.01, *** *p*  <  0.001, **** *p*  <  0.0001, and ns: non-significant. Control: Untreated macrophage; LPS: Lipopolysaccharide-stimulated macrophage; Dex: Dexamethasone; 7OCT: 7-octenoic acid.

To evaluate the anti-inflammatory efficacy of 7OCT, RT-qPCR was employed to quantify the mRNA expression levels of key pro-inflammatory genes in THP-1-derived macrophages following LPS stimulation (Figure 6A). Cells were treated with (concentrations of 50, 80, and 100 μg/mL) or without 7OCT. The analysis demonstrated that 7OCT at all tested concentrations effectively suppressed the expression of the *NFKB1* and *NOS2* genes, suggesting a consistent inhibitory impact on the NF-κB signaling axis and nitric oxide synthesis. In contrast, the expression of *PTGS2*, which encodes cyclooxygenase-2, was significantly downregulated at 50 and 80 μg/mL, whereas treatment at 100 μg/mL did not result in a comparable reduction, implying a possible threshold effect or regulatory feedback at higher concentrations. These results collectively indicate that 7OCT exerts anti-inflammatory effects by modulating transcriptional activity of pivotal inflammatory mediators in LPS-challenged macrophages.

To explore the impact of 7OCT on the NF-κB signaling pathway, Western blot analysis was conducted in THP-1 macrophages stimulated with LPS. The findings indicated that treatment with 7OCT at concentrations of 50, 80, and 100 μg/mL led to a reduction in phosphorylated IκB-α and COX-2 protein levels (Figure 6B,C). To further assess whether 7OCT modulates the expression of pro-inflammatory proteins through regulation of NF-κB activation, we examined the nuclear translocation of the p65 subunit, a hallmark of NF-κB pathway activation. The distribution of p65 protein between cytosolic and nuclear compartments was evaluated in cells treated with or without LPS, in the presence or absence of 7OCT (Figure 6D). Upon LPS stimulation, a pronounced increase in nuclear p65 levels was observed, indicative of NF-κB activation, whereas 7OCT treatment effectively mitigated this translocation, suggesting its ability to inhibit NF-κB nuclear activity.

### 2.5. The Effect of 7OCT on NADPH Oxidase 2 (NOX2) Pathway in LPS-Stimulated Macrophages

**Figure 7:**
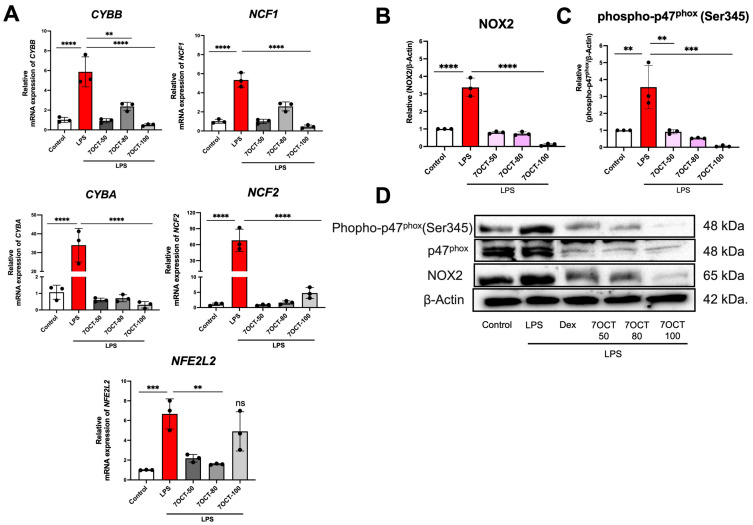
The influence of 7OCT on the expression of oxidative stress-related genes and proteins. RT-qPCR was used to assess the mRNA levels of *CYBB*, *CYBA*, *NCF1*, *NCF2*, and *NFE2L2* (**A**). Western blot analysis was performed to determine the protein expression of NADPH oxidase 2 (NOX2) and phosphorylated p47^phox^ (Ser345) (**D**). (**B**,**C**) Densitometric analysis of bands in D. Results represent the mean ± SEM. A value of *p*  <  0.05 was considered to be significant. Significant differences between groups are marked with: ** *p*  <  0.01, *** *p*  <  0.001, **** *p*  <  0.0001, and ns: non-significant. Control: Untreated macrophage; LPS: Lipopolysaccharide-stimulated macrophage; 7OCT: 7-octenoic acid.

Oxidative stress arises from a disruption in the balance between reactive oxygen species (ROS) generation and the antioxidant defense systems. To investigate this, the expression levels of genes and proteins associated with ROS regulation were examined (Figure 7). LPS stimulation led to an upregulation of CYBB, CYBA, NCF1, NCF2, and NFE2L2 genes encoding components of the NADPH oxidase complex, a major source of ROS. However, this LPS-induced expression was effectively inhibited in cells pretreated with 7OCT at 50, 80, and 100 μg/mL, indicating the compound’s ability to suppress oxidative stress at the transcriptional level.

### 2.6. The Effect of 7OCT on Apoptosis in LPS-Stimulated Macrophages

**Figure 8:**
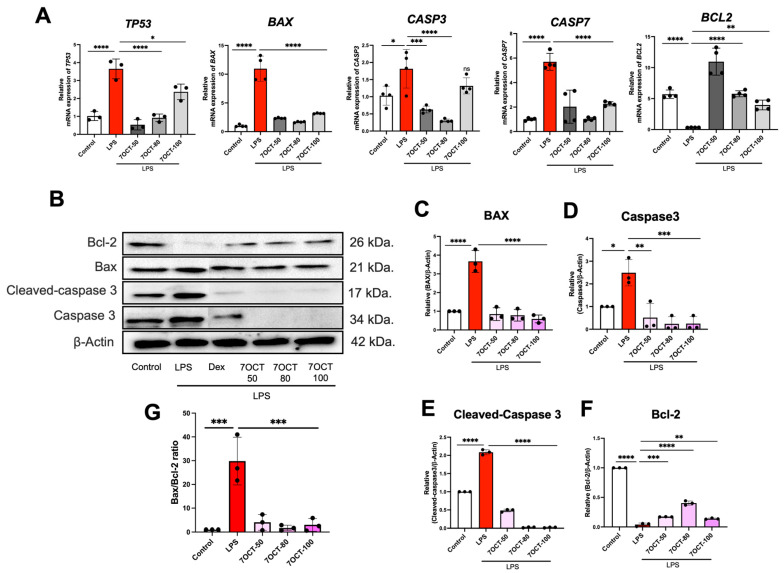
Evaluation of 7OCT’s impact on apoptosis-related gene and protein expression. mRNA levels of TP53, BAX, CASP3, CASP7, and BCL2 were quantified through RT-qPCR (**A**). Western blotting of Bax, Caspase 3, Cleaved-caspase 3, and Bcl-2 (**B**). (**C**–**F**) Densitometric analysis of bands in (**B**). (**G**) Bax/Bcl-2 ratio. β-actin proteins were used as housekeeping proteins. Results represent the mean ± SEM. A value of *p*  <  0.05 was considered to be significant. Significant differences between groups are marked with: * *p*  <  0.05, ** *p*  <  0.01, *** *p*  <  0.001, **** *p*  <  0.0001, and ns: non-significant. Control: Untreated macrophage; LPS: Lipopolysaccharide-stimulated macrophage; 7OCT: 7-octenoic acid.

Given the critical role of apoptosis in the progression of inflammation, the anti-apoptotic potential of 7OCT was assessed by examining gene expression profiles using RT-qPCR. THP-1-derived macrophages were stimulated with LPS and subsequently treated with 7OCT at concentrations of 50, 80, and 100 μg/mL. LPS exposure led to a marked increase in the transcription of TP53, BAX, CASP3, and CASP7, while simultaneously downregulating the anti-apoptotic gene BCL2 (Figure 8A). Treatment with 7OCT resulted in a notable suppression of pro-apoptotic gene expression, including p53, Bax, Caspase-3, and Caspase-7, but an increase in BCL2, indicating its capacity to attenuate apoptosis at the molecular level in LPS-challenged macrophages (Figure 8).

Potential downregulation of apoptosis marker proteins: Western blot analysis of Bax, Caspase-3, Cleaved Caspase-3, and Bcl-2 is shown in Figure 7. LPS stimulation led to the upregulation of Bax, Caspase-3, and Cleaved Caspase-3 (Figure 8C–F). In contrast, Bcl-2 was downregulated in the LPS-only treated group. Treatment with 7OCT resulted in the upregulation of the anti-apoptotic protein Bcl-2, which was associated with the inhibition of the apoptosis marker proteins Bax, Caspase-3, and Cleaved Caspase-3 (Figure 8F). Western blot analysis showed that LPS stimulation significantly increased the BAX/BCL-2 ratio compared with the control group, indicating an enhancement of pro-apoptotic signaling. Treatment with 7oct at concentrations of 50, 80, and 100 µM markedly reduced the BAX/BCL-2 ratio, suggesting suppression of apoptosis (Figure 8G).

## 3. Discussion

The molecular docking results suggested that 7-octenoic acid (7OCT) may interact with several proteins implicated in inflammatory, oxidative, and apoptotic pathways. For inflammation-related targets, including TLR4, NF-κB p65, p50, IκB-α, and COX-2, 7OCT displayed weak-to-moderate predicted binding affinities, with slightly higher scores observed for NF-κB p65 and COX-2. These observations are hypothesis-generating, indicating potential interactions that could influence key signaling cascades associated with inflammation, but they do not establish direct causal inhibition. Similarly, docking analyses with NOX2 subunits (p22^phox^, p47^phox^, p40^phox^, and p67^phox^) suggest possible involvement in modulating reactive oxygen species (ROS) production, though these predictions remain speculative [15], In the context of apoptosis, 7OCT showed moderate predicted affinity for pro-apoptotic proteins such as BAX and Caspase-3, as well as for the anti-apoptotic protein Bcl-2, highlighting possible effects on the balance between cell survival and programmed cell death [27,28]. Overall, these docking results provide preliminary, supportive insights that may guide further experimental studies but should be interpreted cautiously. They complement our experimental findings and generate testable hypotheses regarding the potential anti-inflammatory, antioxidant, and anti-apoptotic properties of 7OCT, which require validation in vitro and in vivo models.

Lipopolysaccharide (LPS), a widely recognized bacterial endotoxin, elicits potent activation of the innate immune system by triggering the release of pro-inflammatory cytokines such as IL-6, IL-1β, and TNF-α [41,42]. These mediators play pivotal roles in orchestrating inflammatory signaling and recruiting immune cells to sites of infection or tissue injury. Beyond cytokine induction, LPS engagement activates transcriptional programs that drive inflammation, including upregulation of genes such as *NFKB1, PTGS2*, and *NOS2*, which encode critical components like NF-κB p50, cyclooxygenase-2 (COX-2), and inducible nitric oxide synthase (iNOS), respectively. At the protein level, LPS stimulation is characterized by enhanced NF-κB pathway activity, as evidenced by nuclear translocation of the NF-κB p65 subunit, phosphorylation and subsequent degradation of its inhibitor IκB-α, and increased expression of COX-2. These molecular events collectively reflect a robust inflammatory cascade [43,44,45]. In this context, treatment with 7OCT significantly attenuated the LPS-induced expression of IL-6, IL-1β, and TNF-α, underscoring its immunomodulatory potential. Furthermore, 7OCT suppressed the transcription of *NFKB1, PTGS2*, and *NOS2*, indicating that it may interfere with upstream inflammatory transcriptional regulators. Protein-level analysis supported these findings, revealing that 7OCT reduced IκB-α phosphorylation, impeded NF-κB p65 nuclear translocation, and diminished COX-2 expression. These observations suggest that 7OCT targets multiple nodes within the NF-κB signaling axis, thereby suppressing both the initiation and propagation of inflammatory responses. Together, these in vitro and in silico results provide compelling mechanistic evidence supporting the anti-inflammatory efficacy of 7OCT and affirm its potential as a therapeutic agent in inflammation-associated pathologies.

Antioxidant defenses are intrinsically linked to inflammation, as unchecked reactive oxygen species (ROS) generated during inflammation can propagate tissue injury. A central ROS source is the enzymatic NADPH oxidase 2 (NOX2) complex, which responds to inflammatory triggers like LPS by transferring electrons from NADPH to molecular oxygen to generate superoxide. Although this oxidative burst is part of pathogen defense, chronic or excessive activation leads to oxidative stress and cellular damage. NOX2 is composed of both membrane-bound subunits CYBB (gp91^phox^) and CYBA (p22^phox^) and cytosolic regulatory subunits, including NCF1 (p47^phox^) and NCF2 (p67^phox^). Upon activation, p47^phox^ is phosphorylated, enabling its translocation and assembly with the membrane complex, thereby facilitating ROS production [46,47,48]. In our study, 7OCT exerted a potent antioxidant effect by suppressing LPS-induced oxidative stress at both transcriptional and translational levels. Treatment with 7OCT markedly downregulated mRNA levels of *CYBB*, *CYBA*, *NCF1*, *NCF2*, and *NFE2L2* (which encodes the redox-regulator Nrf2), thereby impairing NOX2 complex formation and attenuating ROS generation. Furthermore, 7OCT decreased the protein levels of NOX2 and phosphorylated p47^phox^, blocking the post-translational modifications essential for NOX2 activation. This dual-action mechanism targeting both gene expression and protein activation underscores 7OCT ’s efficacy as an antioxidant agent capable of mitigating oxidative stress related to inflammation.

While 7OCT, a compound derived from Moringa leaves, is known for its anti-inflammatory and antioxidant properties, its role in modulating apoptosis also holds therapeutic potential. Our findings revealed that treatment with 7OCT led to increased expression of the anti-apoptotic gene Bcl-2, along with a concurrent reduction in the expression of key pro-apoptotic markers, including Bax and caspase-3. Moreover, we observed that p53, a transcriptional activator of Bax, was significantly suppressed following LPS stimulation in the presence of 7OCT. These observations indicate that 7OCT may exert a protective effect against LPS-induced apoptosis by interfering with the p53-mediated apoptotic cascade, particularly through inhibition of the Bax and caspase-3 signaling axis. 7OCT resulted reduction of the BAX/BCL-2 ratio, suggesting that 7oct exerts an anti-apoptotic and cytoprotective effect against LPS-induced apoptosis reduction of the BAX/BCL-2 ratio, suggesting that 7oct exerts an anti-apoptotic and cytoprotective effect against LPS-induced apoptosis

Our finding presents novel evidence that 7OCT effectively counteracts oxidative stress by disrupting the LPS-induced activation of NADPH oxidase 2 (NOX2), achieved through the downregulation of its key subunit proteins responsible for reactive oxygen species (ROS) generation. In addition to its antioxidant action, 7OCT was found to significantly suppress the NF-κB signaling cascade, a central pathway in the regulation of inflammation. The compound also exhibited anti-apoptotic effects by diminishing the expression of TP53, a gene known to regulate the pro-apoptotic marker BAX. Collectively, these results underscore the multi-targeted biological activity of 7OCT, encompassing anti-inflammatory, antioxidant, and anti-apoptotic mechanisms. These findings suggest that 7OCT is a promising candidate for the development of future therapeutic agents aimed at mitigating inflammatory conditions. Although the precise quantity is unknown, the natural food *Moringa oleifera* Lam. leaves contain 7OCT. Nonetheless, further validation through in vivo and clinical research is essential to realise its pharmacodynamics and therapeutic potential.

## 4. Materials and Methods

### 4.1. Cell Differentiation and Culture

THP-1 cells were maintained in RPMI 1640 medium (Gibco, Carlsbad, CA, USA) supplemented with 10% fetal bovine serum (FBS) (Gibco, Carlsbad, CA, USA) and 1% penicillin/streptomycin (Gibco, Carlsbad, CA, USA) under standard conditions of 5% CO_2_ and 37 °C. For differentiation, THP-1 cells at a density of 2 × 10^5^ cells/mL were treated with 100 nM phorbol 12-myristate 13-acetate (PMA, Sigma-Aldrich, St. Louis, MO, USA) for 24 h. After this initial PMA exposure, the medium containing PMA was replaced with fresh complete RPMI 1640, and cells were further cultured for an additional five days to promote full differentiation.

### 4.2. Cytotoxicity Assessment by MTT Assay

THP-1-derived macrophages (2 × 10^4^ cells/mL) were cultured in complete media as described above and maintained at 37 °C with 5% CO_2_. The investigation of bioactive compounds in *Moringa oleifera* within THP-1-derived macrophages was carried out using the MTT assay. In brief, THP-1-derived macrophages were stimulated with varying doses of 7OCT (Sigma-Aldrich, St. Louis, MO, USA, Cat: 715751, purity (≥97%)) in a co-solvent system of DMSO: Tween 80 (1:1, *v*/*v*) for 24 h. Subsequently, the cells were washed twice with media, and 100 μL of 1 mg/mL MTT reagent was added. After incubation for 3 h at 37 °C with 5% CO_2_, the absorbance signals were measured at 570 nm using a Varioskan LUX Multimode Microplate Reader (PerkinElmer, Inc., Waltham, MA, USA). Untreated cells served as the control.

### 4.3. Enzyme-Linked Immunosorbent Assay (ELISA)

To investigate the effects of 7OCT on inflammatory responses, THP-1-derived macrophages were plated at 0.5 × 10^6^ cells per well in 12-well plates and incubated for 24 h. These cells were then subjected to a 1-h pre-treatment with varying concentrations of 7OCT (50, 80, and 100 μg/mL) or with 100 nM dexamethasone. Subsequently, an inflammatory response was induced by treating the cells with 1 μg/mL of LPS for 24 h. Control wells remained untreated. We quantified the concentrations of pro-inflammatory cytokines, namely human IL-6 (Sino Biological, Cat: SEKB10395, Beijing, China), human IL-1β, and TNF-α (Sino Biological, Cat: SEKA10602, Beijing, China), in the cell culture supernatants using ELISA kits as per manufacturer protocols. Absorbance (450 nm) readings were obtained utilizing a Varioskan LUX Multimode Microplate Reader (PerkinElmer, Inc., Waltham, MA, USA).

### 4.4. Reverse Transcription Quantitative Real-Time PCR (RT-qPCR)

THP-1-derived macrophages were seeded into 12-well plates at 0.5 × 10^6^ cells/well and incubated for 24 h. Cells then received a 1-h treatment with either 7OCT (50, 80, or 100 μg/mL) or 100 nM dexamethasone. Following this, an inflammatory challenge was initiated by co-treating cells with the same samples and 1 μg/mL of LPS for 24 h. Total RNA isolation was performed using Trizol reagent (Invitrogen, Carlsbad, CA, USA) as per the manufacturer’s instructions, followed by cDNA synthesis with the Tetro cDNA Synthesis Kit (BIOLINE USA Inc., Taunton, MA, USA). Gene expression differences between groups were determined via real-time PCR on a CFX96 Touch Real-Time PCR Detection System (Bio-Rad, Hercules, CA, USA). Raw quantification cycle (Cq) values were normalized to the β-actin housekeeping gene to derive ΔCq values, which were then converted to fold-changes relative to the control group using the 2^−ΔΔCq^ method. Table 2 lists the primers used in this investigation.

### 4.5. Western Blot Analysis

For protein analysis, THP-1-derived macrophages were cultured in 12-well plates at a density of 0.5 × 10^6^ cells per well for 24 h. Subsequently, cells were pre-treated for 1 h with either 7OCT (50, 80, and 100 μg/mL) or 100 nM dexamethasone. An inflammatory stimulus was then applied by co-treating cells with the same samples and 1 μg/mL of LPS for an additional 24 h. Whole-cell protein lysates were prepared by lysing cells in RIPA buffer supplemented with a Protease/Phosphatase Inhibitor Cocktail for 30 min, followed by centrifugation at 14,000× *g* for 15 min at 4 °C. For nuclear protein extraction, cells were first subjected to cytoplasmic/nuclear fractionation. The resulting nuclear pellet was resuspended in a high-salt buffer, incubated on ice with intermittent vortexing, and then centrifuged at 14,000× *g* for 15 min at 4 °C to yield the nuclear protein supernatant. Protein concentrations were determined using the Bradford assay. Samples were then resolved by 12% SDS-PAGE (150 V for 1 h) and electroblotted onto PVDF membranes using a Trans-Blot^®^ Turbo™ Transfer System (Bio-Rad, Hercules, CA, USA) at 25V for 30 min. After washing, membranes were blocked with 2% BSA in TBST (Tris-buffered saline with 0.5% Tween 20) for 1 h at room temperature. Primary antibody (dilution 1:1000) incubation occurred overnight at 4 °C. Following three washes with TBST, membranes were incubated with secondary antibodies (dilution 1:10,000) for 1 h at room temperature. Finally, protein bands were visualized using a horseradish peroxidase chemiluminescence substrate, and band intensities were quantified with Image Lab software (Version 5.1, Hercules, CA, USA).

### 4.6. Molecular Docking Analysis

This study aimed to computationally predict the binding interactions between a specific bioactive compound (7OCT) and target proteins implicated in inflammation, oxidative stress, and apoptosis. The three-dimensional structure of 7OCT (PubChem CID: 543977) was acquired from the PubChem database for use as the ligand. Correspondingly, the crystal structures of the selected inflammatory, oxidative stress, and apoptotic proteins were retrieved from the Protein Data Bank (PDB) to serve as receptors. Prior to docking, receptor proteins were prepared using BIOVIA Discovery Studio Visualizer version 21.1.0.20298, including the addition of hydrogen atoms. To ensure the reliability and accuracy of the molecular docking simulations, a rigorous validation protocol was implemented using AutoDock Tools version 1.5.7 (The Scripps Research Institute, La Jolla, CA, USA). This critical step involved a re-docking procedure: the co-crystallized ligand (from the PDB entry, if available) was separated from its protein, and both files were converted to AutoDock-compatible formats (e.g., .pdbqt). A grid box was then defined with appropriate X, Y, and Z coordinates, along with dimensions designed to encompass the known binding site of the original ligand. Following the re-docking simulation, the predicted pose of the ligand was compared to its experimentally determined crystal structure, with the Root Mean Square Deviation (RMSD) serving as the primary metric for evaluating protocol accuracy. An RMSD value below 2.0 Å was considered indicative of a reliable docking protocol, suitable for further investigation. Conversely, RMSD values exceeding this threshold necessitated recalibration of docking parameters, such as grid box size or the number of docking runs, to improve alignment with experimental data. Subsequent to this validation, the molecular docking simulations with compound 7OCT were performed using AutoDock Vina software version 1.2.3, following a similar procedural pipeline as the validation protocol. Finally, the resulting protein-ligand interactions and potential binding pockets were comprehensively analyzed and visualized using BIOVIA Discovery Studio Visualizer software version 21.1.0.20298 (Dassault Systèmes, San Diego, CA, USA).

### 4.7. Statistical Analysis

Data are presented as the mean ± standard error (SEM) of three independent experiments. For comparisons involving more than two groups, one-way ANOVA was performed with multiple comparison correction (Dunnett test) using GraphPad Prism 10 software. *p*-values less than 0.05 were considered statistically significant.

## 5. Conclusions

These findings reveal that 7OCT has the capacity to mitigate LPS-induced activation of macrophages through a combination of mechanisms. We observed a significant reduction in various inflammatory mediators, including IL-6, IL-1β, TNF-α, iNOS, and COX-2. A key mechanism involves 7OCT’s inhibition of the NF-κB pathway, demonstrated by decreased levels of the p65 transcription factor. Moreover, 7OCT reduces oxidative stress by downregulating the expression of p22phox, p47phox, and p67phox, crucial components of NADPH oxidase 2 (NOX2) involved in free radical generation. Additionally, 7OCT’s protective role against cell death is evident from its ability to decrease pro-apoptotic proteins like Bax and caspase-3, while concurrently elevating the anti-apoptotic protein Bcl-2. These findings collectively underscore 7OCT’s promising anti-inflammatory, antioxidant, and anti-apoptotic properties. To thoroughly understand its full scope in these capacities, in vivo binding validation and further experimental investigations are essential.

## Figures and Tables

**Table 1:** Energy profiling of 7OCT, as ligands docked to protein receptors.

Amino Acid Residues	Docking Score (Kcal/mol)	Protein Name	Compound
HIS207, HIS388, LEU391, THR206	−5.5	Cyclooxygenase-2 (COX-2)	7-Octenoic acid
ASN186	−5.0	v-rel avian reticuloendotheliosis viral oncogene homolog A (RelA)	
ASP136, ILE175, LEU115, LEU130, LEU163, PRO137	−4.6	Inhibitor of nuclear factor kappa-B alpha (IκB-α)	
LYS275, PHE295	−4.5	Nuclear factor NF-κB p50 Subunit (p50)	
PHE377, TYR403	−3.7	Toll-like receptor 4 (TLR4)	
PHE202, TRP207, TRP337, TYR207	−5.9	NADPH oxidase 2 (NOX2)	
ARG90, PHE14, PHE81, TYR24, TYR26	−5.4	Neutrophil cytosolic factor 1 (p47^phox^)	
ARG90, PHE80, THR84, VAL77	−4.9	Cytochrome b-245 alpha chain (p22^phox^)	
LYS253, PHE27, TYR244	−4.2	Neutrophil cytosolic factor 4 (p40^phox^)	
ARG395, LYS418, THR423	−4.0	Neutrophil cytosolic factor 2 (p67^phox^)	
ASN73, ILE66, IEU70	−5.0	BCL2-associated X, apoptosis regulator (BAX)	
ARG64, ARG207, GLN161, HIS121, PHE256, TRP206, TYR204	−4.8	Caspase 3	
HIS186, ILE189, TRP195, THR7, TYR9	−4.5	B-cell lymphoma 2 (Bcl-2)	
GLN560, MET259, ME559, THR563	−4.2	Caspase 7	

**Table 2:** Primer pairs used in the RT-qPCR analyses.

No	Primer	Description	Forward Primer (5′-3′)	Reward Primer (5′-3′)
1	*NFKB1*	Nuclear Factor Kappa B Subunit 1	AACAGAGAGGATTTCGTTTCCG	TTTGACCTGAGGGTAAGACTTCT
2	*PTGS2*	Prostaglandin-Endoperoxide Synthase 2	CTGGCGCTCGACCATACAG	CGCACTTATACTGGTCAAATCCC
3	*NOS2*	Nitric Oxide Synthase 2	CAGGGTGTTGCCCAAACTG	GGCTGCGTTCTTCTTTGCT
5	*CYBB*	Cytochrome b-245 Beta Chain	GCTGGTGGGTCATCAGGAAA	TGAGCAGCACGCACTGGA
6	*NCF1*	Neutrophil Cytosolic Factor 1	CCTGCAACTACCTTGAACCAGTT	GCCCTGACTTTTGCAGGTACA
7	*NCF2*	Neutrophil Cytosolic Factor 2	CCTGCAACTACCTTGAACCAGTT	GGACTGCGGAGAGCTTTCC
8	*TP53*	Tumor Protein p53	GTGGTAATCTACTGGGACGGA	CTTTCTTGCGGAGATTCTCTTC
9	*BAX*	BCL2-Associated X, Apoptosis Regulator	GGTTGTCGCCCTTTTCTA	CGGAGGAAGTCCAATGTC
10	*CASP3*	Caspase 3	GCTATTGTAGGCGGTTGT	TGTTTCCCTGAGGTTTGC
11	*CASP7*	Caspase 7	ACTGCTCTTGTGCCAAGATG	CATGGCTTAAGAGGATGCAG
12	*BCL2*	B-Cell Lymphoma 2	GATGTGATGCCTCTGCGAAG	CATGCTGATGTCTCTGGAATCT
13	*GAPDH*	Glyceraldehyde-3-Phosphate Dehydrogenase	GGTGGTCTCCTCTGACTTCAACA	GTTGCTGTAGCCAAATTCGTTGT

## Data Availability

All data generated or analysed during this study are included in this published article.
